# Feasibility and Usability of Tele-interview for Medical Residency Interview

**DOI:** 10.5811/westjem.2017.11.35167

**Published:** 2017-12-21

**Authors:** Ali Pourmand, Hayoung Lee, Malika Fair, Kaylah Maloney, Amy Caggiula

**Affiliations:** George Washington University School of Medicine and Health Sciences, Department of Emergency Medicine, Washington, DC

## Abstract

Every year in the United States, medical students and residency programs dedicate millions of dollars to the residency matching process. On-site interviews for training positions involve tremendous financial investment, and time spent detracts from educational pursuits and clinical responsibilities. Students are usually required to fund their own travel and accommodations, adding additional financial burdens to an already costly medical education. Similarly, residency programs allocate considerable funds to interview-day meals, tours, staffing, and social events. With the rapid onslaught of innovations and advancements in the field of telecommunication, technology has become ubiquitous in the practice of medicine. Internet applications have aided our ability to deliver appropriate, evidence-based care at speeds previously unimagined. Wearable medical tech allows physicians to monitor patients from afar, and telemedicine has emerged as an economical means by which to provide care to all corners of the world. It is against this backdrop that we consider the integration of technology into the residency application process. This article aims to assess the implementation of technology in the form of web-based interviewing as a viable means by which to reduce the costs and productivity losses associated with traditional in-person interview days.

## INTRODUCTION

Residency interviews are an important component of the application process to U.S. graduate medical education training programs. Students apply for a residency position in their chosen specialty during the final year of medical school. This process begins with submitting a written application through the Electronic Residency Application Service (ERAS), which is then reviewed by residency program leadership who select of a subset of applicants for on-site interviews over the course of two to three months.^1^ At the end of the interview period, applicants create a rank-order list of programs where they desire to train, and these lists are then submitted to the National Resident Matching Program (NRMP).^2^

The residency program and applicant’s rank-order lists are highly influenced by the interview experience.^3^,^4^ However, the traditional on-site interview process poses a significant resource burden for both applicants and residency programs. The Association of American Medical Colleges (AAMC) reported that median educational debt for medical school graduates in 2015 was over $180,000,^5^ and the degree of debt influences a student’s career planning.^6^ According to the American Medical Association, applicants participate in an average of 12 residency interviews during their final year of medical school.^7^ Often these interviews are not within close proximity to a student’s home institution, thus necessitating costly travel. Concurrently, organizing multiple interview days requires substantial preparation time for residency programs. In addition to financial considerations, travel and preparation time for interviews detracts from medical education and decreases educational and clinical productivity for applicants.

To alleviate some of the financial and productivity burdens of on-site interviews, web-based residency interviews have been proposed as an alternative.^8^–^10^ In this article, we will review the advantages and disadvantages of web-based interviews, analyze their cost effectiveness, and discuss the effect on rank-order lists.

### Advantages and Disadvantage of Web-based Interviews

Traditionally, interviews have been conducted on-site at residency programs in order to engage face-to-face with the applicant and allow them to interact with a variety of current trainees, faculty, and staff. A typical interview day often consists of presentations by program directors and/or department chairs, individual interviews by multiple faculty members, tours, and an optional social event with current residents. Hosting these activities takes considerable coordination with faculty and resident schedules and requires a sizable monetary investment from the residency program. Applicants, in turn, are responsible for financing travel and accommodations for an average of 12 interviews across the U.S.^7^ while maintaining their clinical training. Advantages of web-based interviews include improved scheduling flexibility, reduction of financial burden for residency programs and applicants, and improvement of educational and clinical productivity.

Applicants most commonly decline invitations to interview due to scheduling conflicts, thus reducing the number of programs they can consider when making the rank-order list and decreasing the pool of viable applicants for the program.^8^–^10^ Web-based interviews eliminate travel time and improve flexibility for applicants and residency programs when scheduling interviews. As such, web-based interviews offer residency programs the ability to engage and interview candidates who would otherwise not be able to participate in an on-site interview due to scheduling conflicts.

Along with improved flexibility, eliminating the need for travel also alleviates some financial burden for applicants. In March 2015 the AAMC released a report detailing the expense breakdown of applying to residency programs during the 2014–2015 application cycle.^11^ The total average cost of participating in on-site interviews was $3,422.71 for each applicant. Expenses were significantly higher for applicants who participated in a couples match ($5,506.21) and for those applying to preliminary position programs ($4,575.62). Costs also varied with specialty choice, with neurosurgery residency applicants spending an average of $6,930 and family medicine applicants shouldering the lowest costs at $1,968. According to this report, 79% and 65% of the respondents strongly agreed or agreed that travel and lodging expenses, respectively, were overly burdensome. Furthermore, 58% responded that financial considerations influenced an applicant’s decision to attend interviews.^11^ Therefore, web-based interviews may reduce the impact of financial considerations on the decision to interview at a residency program site.

Web-based interviews may also reduce the financial burden for residency programs. Costs to programs include interview day meals, local transportation between clinical sites, written materials, and staff time dedicated to the interview day.^8^ According to Shah et al. (2011), the average cost for the University of New Mexico’s urology residency program to host an on-site interview was $5,031.68 for each interview process. In contrast, when a web-based interview was conducted, the financial cost of each interview process was significantly lower, averaging $2,159.40.^8^

In addition to the financial benefits of web-based interviews, educational and clinical productivity may improve. Traditional on-site residency interview days decrease time spent dedicated to educational pursuits for applicants and reduce faculty clinical hours. Applicants commit an average of 20 days to residency interviews, time therefore not devoted to medical education.^8^, ^12^ Only 10% of applicants who participated in web-based interview missed one or more days of school, compared to 30% of applicants who participated in on-site interviews (*p = 0.04*).^8^ Faculty members who practice clinically usually conduct residency interviews. Edje et al. and Tempe et al. observed that residency programs using a web-based interview process decreased the total time dedicated to interviews by seven days, thus theoretically increasing clinical work productivity of faculty members.^13^,^14^

Other considerations include number, length, and timing of interviews. The number and duration of interviews can be kept consistent between the two modalities. With regard to scheduling the web-based interviews, applicants can be offered the option to meet in the morning, afternoon, or evening to accommodate time zone differences.^15^ Offering evening interviews allows for fewer interruptions and conflicts with daytime clinical and educational responsibilities for both applicants and faculty.^8^

Despite potential improvements in cost and productivity, some are hesitant to engage in web-based interviews due to perceived disadvantages. Common concerns include an applicant’s inability to interact with current trainees and faculty.^10^ Many also believe that applicants are better equipped to evaluate a city and program during an on-site interview.^10^,^15^,^16^ Healy et al. reported that among the residents who interviewed for an orthopedic fellowship position via web-based interviews, some candidates felt that they either did not have the opportunity to present themselves adequately or did not feel “comfortable enough to rank the program.” It was concluded that using this interview platform adversely affected the program’s position on an applicant’s rank list. This unfavorable outlook can negatively impact a program’s ability to recruit the best applicant as well as the resident’s capacity to find the best programmatic fit.^16^ Conversely, one study indicated that there was no difference in the rank given to applicants by faculty, and tele-interviewing was associated with matching highly ranked applicants to their program.^16^ Although studies have shown that most interviewees were satisfied with their web-based interview experience, little research has been conducted to evaluate how video interviewing affects an applicant’s rank-order list.^10^,^16^

Some disadvantages can be at least partially mitigated through proper planning and structuring of web-based interviews to closely mimic on-site interviews. Typically, programs prepare hard copies of information pertaining to the residency such as curriculum, clinical schedule, resident demographics, faculty biographies, research initiatives, and surrounding community.^17^ These materials can be provided digitally for web-based interviewees. Similarly, presentations given by faculty and staff during on-site interview days can be replaced with recorded videos. On-site hospital tours can be substituted with interactive virtual tours of an institution’s clinical sites, facilities, and surrounding geographic area.^15^ Designing an accurate and informative electronic manual, videos, and tours is crucial to ensuring web-based interviewees receive sufficient information regarding the program, research opportunities, and culture. When an adult reconstruction fellowship program at Newton-Wellesley Hospital offered video tours, 83% of the web-based interviewees found the video tour helpful.^16^ In addition, 85% of the candidates believed that the manual and web-based interview gave them a satisfactory and sufficient understanding of the program, though 17% still chose to visit the hospital after the interview.^16^

Opportunities to interface with current residents or faculty can be offered to web-based interviewees by providing contact information. Although interacting with current trainees was identified as an important factor to decide rank-list order,^1^ only 28% of the adult reconstruction fellowship web-based interviewees contacted a current fellow.

While it is challenging to predict and minimize technological difficulties with online applications, Shah et al. established a protocol that allowed for troubleshooting well in advance of the actual interview. Their team provided written instructions for establishing a software account a month prior to the web-based interview, conducted a test call with the program coordinator to verify a successful connection during the preceding week, and offered faculty members who were unfamiliar with the technology a five-minute tutorial on the day of the interview.^8^ Another potential method to minimize interruptions due to technological failures is to have a technology consultant in the room, thus allowing for immediate access to technical assistance.^16^ Williams et al. also suggested that attention to small and simple details, such as sufficient lighting in the room and proper placement of the camera, made a difference in the quality of the interview.^15^

### Cost Analysis of Web-based Interview

Several studies have investigated the use of technology and web-based interviews as a cost-effective alternative to an on-site interview. The need for additional staff is the most significant financial consideration for the host institution, while travel expenditures account for the greatest cost to applicants.^12^ According to Kerfoot et al. (2008), lodging, food, and clothing accounted for approximately 40% of total applicant expenses, while the remaining 60% was attributed to travel alone.^12^ Table 1 highlights the differences in total costs for on-site versus web-based interviews as demonstrated by several studies.

Edje et al. (2013) analyzed the financial benefits and drawbacks of web-based family medicine residency interviews compared to on-site interviews for both host institutions and applicants during the 2011–2012 application cycle.^13^ According to the post-interview surveys, the cost of a web-based interview for applicants was minimal, especially if the applicant already had access to a microphone and webcam. Therefore, the total financial savings for applicants to participate in a web-based interview was $566 (95% confidence interval: $349 – $784; p < 0.001; t = 5.5826; df = 14; standard error of difference = 101.462).

For residency programs, the total cost of hosting an in-state applicant was $917 compared with $1,027 for an out-of-state applicant.^13^ The authors of the article did not include an expense breakdown but did indicate that the direct salary cost to interviewers was $602 for each on-site applicant. Hosting web-based interviews decreased interviewer expenses to $120 per interview. Furthermore, expenditures related to purchasing and installing the technology necessary for web-based interviews were minimal, totaling only $132.50. Therefore, the program saved approximately $586.40 for each applicant by opting to conduct web-based interviews in lieu of the traditional face-to-face format.^13^

Shah et al. also evaluated the cost effectiveness of web-based interviews compared with on-site interviews for urology residency programs during the 2010–2011 match cycle.^8^ Applicants who accepted the offer to interview were randomly assigned to an on-site or web-based interview. To minimize bias in the selection process, each applicant then underwent a second interview two weeks later – those who had previously interviewed via the Internet would then repeat the process in person and vice versa. The on-site interview consisted of an eight-hour session including breakfast, an interview with the program director, six to eight additional interviews with faculty and chief residents, and a tour of two major teaching facilities. Each interview was 15 minutes long. The web-based process consisted of three to six faculty interviews that lasted approximately 15 minutes, an online tour of the facilities, and an opportunity to ask questions. In addition, there was extensive pre-interview preparation including instruction on the use of the technology a month prior to the interview and a test call to confirm proper functioning of the application.

When considering expenses, it is important to note that the average financial cost for participating in interviews is significantly affected by geography.^12^ Due to the dense distribution of residency programs in the northeastern U.S, applicants from northeastern medical schools have the lowest expenses, averaging $243 per interview. In contrast, applicants from the south spend the most money at an average of $368 per interview. There have been recent advancements in scheduling technology, and some initiatives have been proposed that would allow individual programs to coordinate an applicant’s interviews geographically in an effort to limit travel expenses associated with repeated trips to the same location.^18^ demonstrated that an applicant could theoretically reduce their costs significantly by using such a program, depending on the number of interviews scheduled in a specific area.^18^ While such an initiative would likely provide some cost savings, the overall expenses for applicants are still decreased considerably by participating in web-based interviews by eliminating travel altogether and thus reducing expenditures associated with airfare and accommodations.

### Effect on Rank-order List

Since the interview experience, interaction with residents, and academic reputation are important factors when ranking programs, the impact of web-based interviews on applicant perception of these elements must be considered.^1^ As discussed previously, designing the web-based interview to closely mimic an on-site interview can potentially minimize the difference in the interview experience and the opportunity to interact with residents between on-site and web-based interviews. Subjectively, the tele-interview experience was a positive one for adult reconstruction fellowship applicants at Newton-Wellesley Hospital. Eighty-five percent of applicants believed they were able to adequately represent themselves during the web-based interview, and 81% were comfortable ranking the program.^16^ That said, the study also found that 34% of interviewees believed that the web-based interview had an unfavorable impact on ranking the program.^16^ The reason was not explored in the survey.

The same study also examined the effect of tele-interviewing on the program’s rank list of applicants. After the web-based interview, faculty had the opportunity to meet several of their candidates in person. Neither their opinion of applicants nor rank-list order changed following a face-to-face meeting.^16^ Additionally, after three years of using web-based interviews, authors reported that highly ranked applicants were matched into their program.^16^ The authors of a study looking at the effect of tele-interviewing for ophthalmology resident training at the University of Arizona reported no significant differences in the number of web-based interviewees and on-site interviewees ranked in the top 25 on the program rank list.^19^ The Department of Anesthesiology at Loma Linda University School of Medicine observed that the proportion of applicants accepted to residency programs was not affected by the modality of the interview.^9^

Many prior studies reported subjective data, but only a few discussed decisions on match rank list or admission rate.^9^ The few studies that provide objective data are limited by small sample size and are single-center studies.^8^ Therefore, the impact of web-based interviews on the ultimate decision of rank list and admission rates must be further investigated.

### Applications for Web-based Interview

Another important factor when considering the merits of web-based interviewing is the reliability and usability of available programs and applications needed to facilitate the process. Several studies including Edje et al. and Vadji et al. demonstrated the successful use of free applications such as Microsoft Skype™ and Apple Facetime™.^9^,^13^ With its widespread use (more than 74 million users exist today) and universal video-conferencing applications, Skype is a viable platform for web-based interviews. It supports group/multi-person conferencing, allowing for the applicant and each member of a panel of interviewers to be at different locations. FaceTime boasts similar advantages but is limited in application, being available exclusively on Apple products and restricted to one-to-one video chat.^19^,^20^ However, these programs are not without their drawbacks. Sullivan et al. found that familiarity and ease with Skype and Facetime varied depending on age, and the older generation may not have or want access to these applications.^21^ Furthermore, studies have shown that there are occasional delays in both audio and video, up to 100ms, leading to disjointed calls that can negatively impact the interview process.^21^

Paid programs are also available to facilitate these interviews, such as Cisco WebEx™, which can be used for telephone or video conferencing. Minimum requirements include an account at the hosting institution, an Internet connection, and a computer with a camera (preferably 720p or better). The interviewee must have access to an email address to receive a link to join the conference. Moreover, these programs are capable of conducting tests to determine speed and connectivity prior to the interview, which will in turn affect picture and sound clarity. This and similar programs require Internet speeds of at least five megabits per second for a 720p camera or 1.3 mbps for lower resolutions. Table 2 demonstrates the minimum system requirements that would support teleconferencing programs such as WebEx.^22^ The obvious disadvantage of these programs is cost, since free options do exist. However, with prices as low as $100 dollars per year, they still allow for financial savings when compared with in-person interviews.^20^,^22^

### How to use video-conferencing programs for interviews

In order to use programs available for web-based interviews, knowledge of their functionality is essential. For programs such as WebEx™, an email sent by the host institution to the applicant will contain a link that enables the interviewee to access the platform, at which point they will be required to entire their name and email address. Four connectivity options are available including “Call me,” “I will call in,” “Call using computer,” or “call my video system.” The first two allow for audio-only conferencing. “Call me” and “Call using computer” are available if a mobile device is being used.^22^ For applications such as Skype and FaceTime, all parties involved must have an account. With regard to Skype, the applicant can then add the host institution’s account to his contact list, and either party can initiate a call. With group calls or panel interviews, up to six participants form a group on the application, and then the entire group is connected simultaneously using the video call button.^23^

An iOS device such as an iPhone, iPad, or Mac computer is required for the FaceTime application. If an iPhone or iPad is used, an Apple ID account is required, and the participant must be signed in at the time of use. When accessing the application via a Mac computer, FaceTime can be used without signing into an account. The email address or phone number of the party being called is then entered in manually, and the call can be initiated.^10^ There are also various free applications such as Viber, WhatsApp, Telegram, and imo that could run on Android or Apple products.

## DISCUSSION

While interviews are an integral part of creating the rank list for both applicants and residency programs, traditional on-site interviews can involve significant scheduling conflicts, financial burden, and reduced productivity. Some of these challenges may be alleviated when using a web-based approach to interviewing.

Advancement in high-speed Internet and technology has revolutionized communication, productivity, and efficiency. Furthermore, technology continues to enable the growth of new and innovative ways to practice medicine. Telemedicine increases access and convenience and reduces the cost of healthcare delivery.^24^ Videoconferencing is frequently used in graduate and continuing medical education.^25^,^26^ The AAMC has recently introduced a resource guide for standardized video interview operational pilot, discussing how to register, interview policies, rules to protect interview integrity, and post-interview procedures.^27^

Before web-based interviews are incorporated universally as an efficient alternative to on-site interviews, additional studies must evaluate the potential risk to students whose web-based interview may impose a bias that could be eliminated in person. For example, some applicants’ home environments may not be appropriate for a professional interview. Additionally, students may not have access to the advanced technology required for these Internet applications. Medical schools can consider creating interview rooms on campus in order to standardize the virtual interview experience for their students. Furthermore, studies should explore whether students of various geographic regions, ethnicities, or socioeconomic groups are more or less likely to participate in a web-based interview and the subsequent impact on rank-order lists and matching rates.

## CONCLUSION

In summary, web-based interviews are cost effective for applicants and residency programs. They reduce scheduling conflicts, thus potentially increasing the qualified applicant pool, and they decrease interruptions to educational pursuits and clinical responsibilities. Both financial considerations and time constraints pose significant challenges for applicants and residency programs when accommodating on-site interviews. While the actual cost savings may differ depending on specialty, structure of interviews, geographic location, and the number of applicants, web-based interviews have been shown to be cost-effective compared to traditional practices. More studies should be done to further evaluate the viability of Internet interviews as an alternative option.

## Figures and Tables

**Table 1:** Cost analysis for web-based interview of residency applicants.

	Study	Residency	On-site	Web-based	Savings
Cost analysis for applicants	Edje et al. (2013)	Family medicine	-	Minimal	$566*
	Kerfoot et al. (2008)	Urology	Ave = $330/interview; Northeast: $243 Midwest: $300 West: $333 South: $368	-	-
	Shah et al (2011)	Urology	$364 ± 184 (0–800)**	$171 ± 229 (0–600)**	$193
Cost analysis for residency programs	Edje et al. (2013)***	Family medicine	$917 – $1027	$132.50	$586.40
	Shah et al. (2011)****	Urology	$5,031.68	$2,159.40	$2,872.28

* 95% CI: $349 – $784; p < 0.001; t = 5.5826; df = 14; standard error of difference = 101.462

** p = 0.05

*** Expenses per applicant.

**** Expenses per interview day.

**Table 2:** Basic requirement for online access^20^ to conduct web-based interviews.

	Windows	Mac OS X
Operating system	Windows 7 and above (32 bit/64 bit)	10.7 and above
Processor	Intel Core2 Dup CPU 2.XXGhz or AMD processor with 2 GB of RAM recommended	Interlude (512 MG of RAM or more)
Browsers		
Safari		5–8
Firefox	50.0 ( ^*^ the 64 bit is not supported)	50.0
Internet Explorer	7 and up	
